# Increased intra-thalamic and thalamo-cortical functional connections during human REM sleep: Insights from a two-night EEG-fMRI study

**DOI:** 10.1162/IMAG.a.1026

**Published:** 2025-11-20

**Authors:** Fan Nils Yang, Dante Picchioni, Jacco A. de Zwart, Peter van Gelderen, Jeff H. Duyn

**Affiliations:** Advanced MRI Section, Laboratory of Functional and Molecular Imaging, National Institute of Neurological Disorders and Stroke, National Institutes of Health, Bethesda, MD, United States

**Keywords:** REM sleep, thalamus, functional MRI, human nocturnal sleep

## Abstract

Rapid Eye Movement (REM) sleep, characterized by vivid dreaming and muscle atonia, raises the fascinating question about how such immersive experiences occur without external sensory input. To investigate this, we analyzed data from a whole-night 8-hour EEG-fMRI concurrent recording study with 12 non-sleep-deprived participants. Our findings confirmed that during REM sleep, all thalamic subnetworks simultaneously connect with multiple sensory networks, including visual, motor, auditory, and action-planning networks, which was not observed during wake. Additionally, intra-thalamic connection was significantly higher during REM sleep compared to wake in all 11 participants with high-quality fMRI data during REM, and compared to Non-REM stages in nine out of 11 participants. We validated these results on seven participants who achieved REM sleep on the first (adaptation) night. Building on these findings, we further hypothesized that the observed increases in thalamus-related FCs were specifically linked to phasic REM, a state associated with vivid dreaming, as opposed to tonic REM. Using the Hidden Markov Model to classify phasic and tonic REM states, we found that phasic REM exhibited significantly greater intra-thalamic and thalamo-cortical FCs than tonic REM. These results suggest that during REM sleep, specifically during phasic REM, thalamic subnetworks function collectively to distribute internally generated sensory information to sensory-related cortical networks, which might be the neural mechanisms underlying vivid dreaming.

## 1 Introduction

Rapid Eye Movement (REM) sleep, marked by rapid eye movements, vivid dreaming, and muscle atonia—temporary muscle paralysis preventing physical responses to dreams—is a vital phase of the human sleep cycle. It is often referred to as a “paradoxical” sleep stage because, while the body is in a state of muscle atonia, the brain exhibits activity patterns similar to wake. Accounting for 20–25% of nocturnal sleep, REM sleep plays a critical role in physical, cognitive, and emotional health, and memory consolidation (Boyce et al., 2017; Simor et al., 2020). Despite REM’s significance, we know little about the neural mechanisms of this paradoxical sleep stage.

Functional magnetic resonance imaging (fMRI) is a non-invasive brain imaging technique that has successfully been used to study the neural mechanisms of REM sleep (Chow et al., 2013; Hong et al., 2009; Miyauchi et al., 2009). However, the fMRI studies of REM sleep are sparse because of the following reasons: a) fMRI scanning generates loud acoustic noises, b) fMRI requires maintaining a supine body position and stable head position for the full scan duration, c) capturing REM sleep requires long scan sessions as it typically takes time to establish, that is, REM sleep typically happens 90 minutes after falling asleep and occurs more often toward the second half of sleep, and d) concurrent EEG recording is needed to perform sleep scoring. To overcome these challenges, we established and validated procedures for recording all-night EEG-fMRI in sleeping subjects without sleep deprivation by carefully leveraging core principles and techniques from sleep and neuroimaging research (Moehlman et al., 2019). The resulting dataset offers us an unprecedented opportunity to study the neural mechanisms of nocturnal REM sleep in humans.

REMs are generally thought to be visually guided saccades that reflexively scan and explore the visual scenes generated within dreams (Arnulf, 2011; Hong et al., 1997). Consistent with this, previous fMRI studies found REM-time-locked activation in several brain regions that included visual cortex and thalamus (Hong et al., 2009; Miyauchi et al., 2009). Interestingly, one of these studies also reported activations in multiple non-visual primary sensory cortices (Hong et al., 2009). The simultaneous activation of visual and non-visual sensory cortices during REM sleep is intriguing, as it occurs in the absence of external sensory input during sleep. These findings indicate that during REM sleep, the thalamus functions as an integrated unit, exhibiting simultaneous functional connections (FCs, i.e., interregional signal correlations) with multiple sensory cortices. In contrast, during wake, Seitzman et al. (2019) defined 12 thalamic regions of interests (ROIs) that can be grouped into 5 different subnetworks based on their FCs with cortical networks (Seitzman et al., 2019), including default mode network (DMN), visual network (VIS), auditory network (AUD), lateral and dorsal somatomotor network (lSMN and dSMN). It should be noted that the majority of thalamic nuclei, for example, intralaminar and medial thalamus, receive little sensory input (Saalmann, 2014). Therefore, we hypothesize that these thalamic subnetworks function collectively as a sensory relay station, distributing internally generated sensory information to various sensory cortices/networks and contributing to the vivid dream experiences characteristic of REM sleep. This hypothesis is now testable due to two key advancements: (1) ongoing improvements in fMRI spatial resolution, surpassing that of earlier REM sleep fMRI studies (Chow et al., 2013; Hong et al., 2009; Miyauchi et al., 2009) and (2) the extended duration of REM sleep captured in the all-night fMRI-EEG recording study described above (Moehlman et al., 2019).

**Fig. 1.**
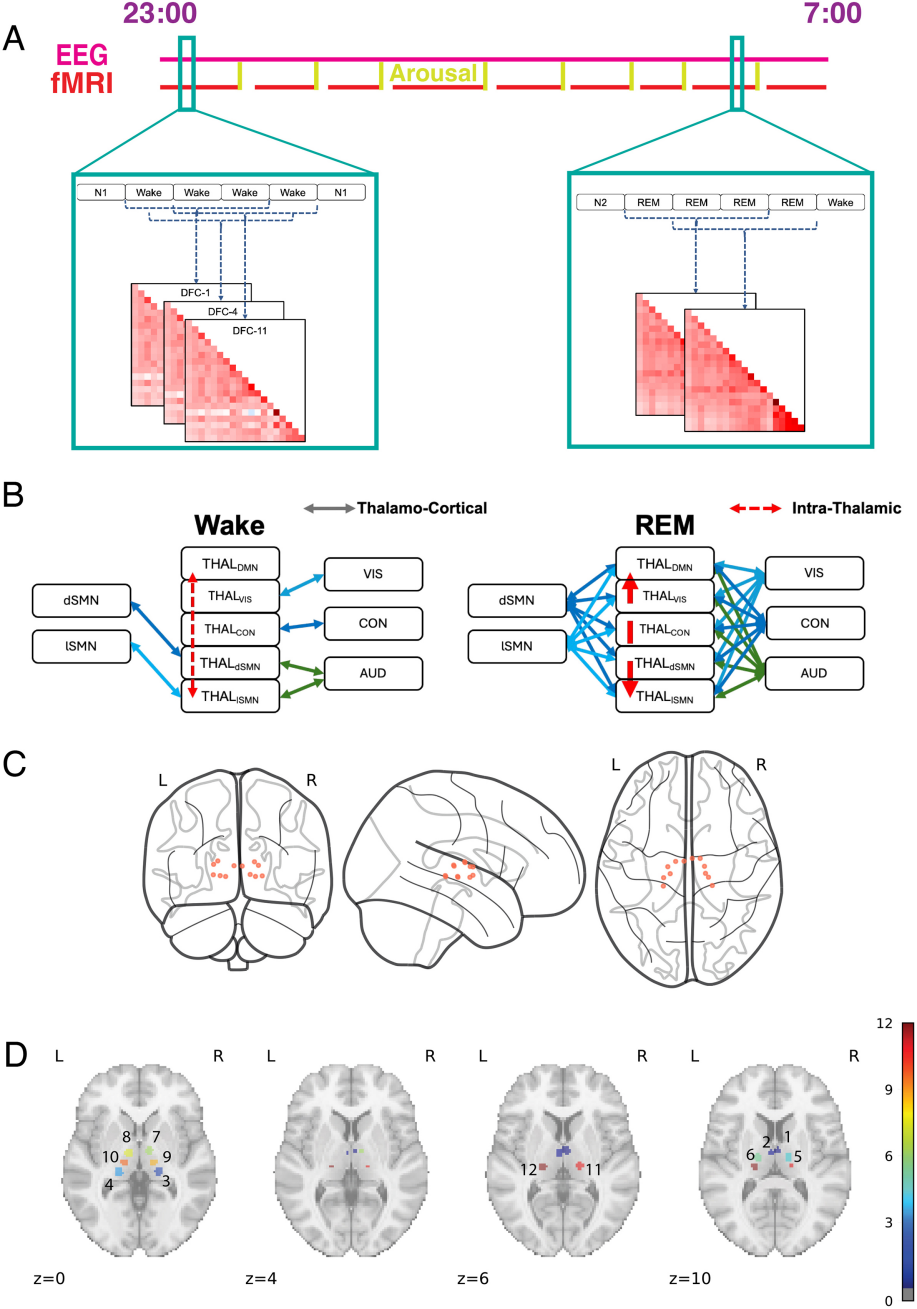
Study design, hypothesis and illustration of Thalamic ROIs. (A) Participants underwent simultaneous EEG-fMRI recording while sleeping inside a scanner from approximately 23:00 to 07:00 over two consecutive nights. Each night, the fMRI sessions were intermittently interrupted by either eight randomly timed acoustic arousals or spontaneous awakenings. Dynamic functional connections were assessed using a sliding window approach during periods of at least three consecutive epochs in the same sleep stage. (B) We hypothesized that during Wake, five thalamic subnetworks have distinct FC patterns. However, during REM sleep, all five thalamic subnetworks would exhibit similar FCs with cortical sensory-related networks, including VIS, CON, dSMN, and lSMN. Additionally, intra-thalamic FC would increase. (C) Twelve thalamic ROIs defined by Seitzman et al. (2019) are displayed. For visualization purposes, the ROI sizes are shown with a diameter of 8 mm, larger than their actual diameter of 4 mm. (D) Mask of 12 thalamic ROIs that were resampled to the functional images with 2 × 2 × 2 mm^3^ resolution. ROI 1-2 are *THAL_DMN_*; 3-4 are *THAL_VIS_*; 5-8 are *THAL_CON_*; 9-10 are *THAL_lSMN_*; and 11-12 are *THAL_dSMN_*. Note: VIS, visual network; AUD, auditory network; CON, cingulo-opercular network, also known as the action-mode network (AMN); dSMN, dorsal Somatomotor network; lSMN, lateral Somatomotor Network; FC, functional connection.

From this hypothesis, we derived the following predictions: (1) During wake, the five thalamic subnetworks will display distinct FC patterns with cortical networks; (2) During REM sleep, these subnetworks will exhibit simultaneous FCs with multiple sensory networks, along with increased intra-thalamic connection within these subnetworks. See the illustration in Figure 1B.

## 2 Methods

### 2.1 Data acquisition and processing

Data collection was part of a previously described sleep experiment (Moehlman et al., 2019; Yang et al., 2024), involving two consecutive nights of simultaneous EEG-fMRI recordings while participants slept in a 3T Siemens Skyra MRI scanner from 23:00 to 7:00 with randomly scheduled arousals, see Figure 1A. See Moehlman et al. (2019) for the detailed design of this experiment. The first night served as an adaptation night. Participants maintained regular sleep patterns for 2 weeks prior to the study, confirmed by wearable devices, and no sleep deprivation protocols were applied during the study. All data used in this study were acquired in compliance with human participants research protocols approved by the National Institutes of Health Combined Neuroscience Institutional Review Board (USA, Protocol Number 16 N-0031), with informed consent obtained from all participants.

The fMRI dataset included whole-brain scans with 50 axial slices, each captured at a spatial resolution of 2.5 mm (2.5 × 2.5 mm² in-plane), with a slice thickness of 2.0 mm and a 0.5 mm gap between slices. Scans were taken at a temporal resolution of 3 seconds (TR, repetition time), with a 90° flip angle and an echo time of 36 ms. Data acquisition was conducted using a multi-slice echoplanar imaging (EPI) sequence in an interleaved order. EEG data were recorded concurrently at a sampling rate of 5 kHz using 64 channels, including two EOG, two EMG, and one ECG channels, ensuring comprehensive scalp coverage. To enhance participant comfort during sleep, three occipital electrodes were repositioned to other locations (Moehlman et al., 2019). An MR-compatible EEG system from Brain Products (Gilching, Germany) was used for these recordings. EEG data collection was synchronized with the MRI scanner’s 10 MHz clock.

In addition, peripheral physiological measurements were collected, including a chest belt for tracking respiratory chest movement and finger photoplethysmography (PPG) for monitoring heart rate and peripheral blood volume. These physiological signals were recorded using a Biopac system with TSD200-MRI and TSD221-MRI transducers, alongside an MP 150 digitizer sampling at 1 kHz, all from Biopac (Goleta, CA, USA). Volume triggers from the MRI scanner were also recorded by the Biopac system to retrospectively align the timing of the peripheral physiological data.

Of the initial 16 subjects recruited, 12 non-sleep-deprived participants (ages 18–35, including 8 females) successfully completed both nights of scanning (approximately 23:00 to 07:00). During each night, fMRI scans were occasionally interrupted by either acoustic stimulations (eight times happened randomly through the night) or spontaneous awakenings. Detailed preprocessing steps for fMRI, EEG, and peripheral physiological data are described in previous work (Moehlman et al., 2019; Picchioni et al., 2022). In brief, a modified version of the ‘afni_proc’ script in AFNI (Cox, 1996) was used, which included steps for outlier removal, detrending, RETROICOR (Glover et al., 2000), peripheral physiological measures removal (Özbay et al., 2019), slice timing correction, motion correction, normalization, registration, censoring (with the Euclidean norm of the first derivative of the six motion parameters set to 0.3 mm as a threshold), resampled to 2 × 2 × 2 mm^3^ spatial resolution, and smoothed to 4 mm FWHM. Previous analyses of this dataset indicated that motion during prolonged sleep scans is comparable to that in shorter resting-state scans and is reduced during deep sleep compared to Wake (Moehlman et al., 2019). The EEG data were corrected for MRI gradient and cardio-ballistic artifacts, then down-sampled to 250 Hz using Brain Vision Analyzer software (Morrisville, USA). Sleep scoring was manually performed on 30-second epochs using a central electrode by a sleep technician following standard AASM criteria, filters, and channel referencing (Berry et al., 2020). Sleep scores and peripheral physiological data were resampled to a 3-second resolution to align with the BOLD signal. Unless explicitly stated otherwise, our analyses primarily focused on data from the second night, as the first night was designated as an adaptation night.

### 2.2 Regions of interest (ROIs)

The Seitzman 300-ROI Altas was used to define regions of interest, including 239 cortical ROIs, 34 subcortical ROIs, and 27 cerebellar ROIs (Seitzman et al., 2019), which were further grouped into 14 functional networks. To study thalamo-cortical connection, we separated all subcortical regions from the 14 cortical networks and grouped these subcortical regions based on their strongest FCs to the 14 cortical networks. For example, the 12 thalamic ROIs covered the medio-dorsal, latero-dorsal, ventro-anterior, ventro-lateral, ventro-posterolateral, and lateral geniculate nucleus-pulvinar regions for each hemisphere, see Figure 1C&D. Based on the Seitzman Atlas, these thalamic ROIs were organized into five subnetworks based on their strongest FC with cortical networks: the Default Mode Network (DMN), Visual Network (VIS), Cingulo-Opercular Network (CON), Lateral Somatomotor Network (lSMN), Dorsal Somatomotor Network (dSMN), referred to as THAL_DMN_, THAL_VIS_, THAL_CON_, THAL_lSMN_, and THAL_dSMN_. Although we did not anticipate changes in functional connectivity between the thalamus and hippocampus across sleep stages, the individual variability in hippocampal anatomy led us to replace atlas-based hippocampal ROIs with participant-specific ROIs derived based on an automated segmentation algorithm (Yushkevich et al., 2015).

### 2.3 Dynamic functional connection (DFC)

Given each sleep episode varies in duration, for example, a Wake episode may last several minutes while an N1 episode may be as brief as 30 seconds, we chose DFC over static functional connectivity. To enable fair comparisons across sleep stages, we standardized the analysis by using a 90-second sliding window, which corresponds to three times the duration of sleep stage scoring epochs. Specifically, for a sleep episode where the same stage lasted longer than 3 epochs (90 seconds), DFC was calculated using the sliding window method (length 30 TR, step 1 TR). The within- and between-network FCs for each sliding window were computed as the mean FC (Hagler et al., 2019; Yang et al., 2022), represented by the Fisher-transformed z-scores of the correlation coefficients, between pairs of ROIs within the same network or between two different networks, respectively.

### 2.4 Phasic versus tonic REM

REM sleep alternates between two sub-stages, phasic and tonic REM, with phasic REM frequently associated with vivid dreaming (Simor et al., 2020). Building on our hypothesis that increased thalamo-cortical and intra-thalamic FCs underpin the experience of dreaming, we predict these connection patterns to be more pronounced in phasic REM compared to tonic REM. To test this, we employed a novel sleep characterization approach on the same dataset from our previous study (Yang et al., 2024). In brief, we applied an unsupervised learning technique, the Hidden Markov Model (HMM, https://github.com/OHBA-analysis/HMM-MAR), to all the BOLD time series (300 ROIs from the Seitzman Altas) from the 2nd night without the inferences with sleep scores and identified 21 HMM states. Each state consisted of a different proportion of sleep stages. We applied a winner-take-all approach, assigning HMM states to the dominant sleep stage identified from the PSG data. There are two distinct REM-related states corresponding to phasic and tonic REM states based on: 1) tonic REM state exhibits higher transition probability to Wake-related states and 2) phasic REM state occurred later during the night compared to tonic REM state. It should be noted that the definition of tonic and phasic REM states was different from the traditional definition of phasic and tonic REM, which are typically based on the frequency of eye movements. See Yang et al. (2024) for the details. We analyzed thalamus-related FCs to compare the HMM-defined phasic and tonic REM states. We did not use EOG to define tonic and phasic REM because the EEG, and thus the EOG, signal quality is compromised by MRI artifacts. However, HMM-based REM states correspond well to both eye movements from video and the PSG-based REM sleep stages, see Supplementary Figure S1 for an example run. There are some differences between HMM-defined REM states and REM sleep episodes used in the current study. First, HMM states have 3 seconds temporal resolution, while REM sleep episodes depend on sleep epochs, which are 30 seconds long. Second, HMM REM states were dominated by REM sleep stages but also contained segments from non-REM sleep stages. The REM sleep episode was defined as three or more successive REM sleep stages.

### 2.5 Statistical analyses

We applied the linear mixed effect model to test the FC differences between Wake and REM stages, and also across different sleep stages, that is, (FC ~ sleep stages + 1|participants). Here, the sleep stage was the fixed effect, and the participant was the random effect. Statistical significance was corrected for multiple comparisons using the family-wise error correction.

For comparisons between HMM states, the z-score was used to determine whether the phasic REM state is an outlier compared to all other states. We used the two-sample t-test to test the differences in the thalamo-cortical connections between the phasic REM and the tonic REM.

## 3 Results

All 12 participants reached REM sleep during the second night. However, REM data from one participant were excluded due to not meeting fMRI quality standards (excessive motion). On average, after quality control, each participant had 45.1 ± 15.1 (see Supplementary Table S1 for the number of each participant across six sleep stages) sleep episodes that lasted longer than 3 epochs in the same stage, comprising 3,426 ± 1,156 points of DFC (see Supplementary Table S2 for the number of each participant across six sleep stages).

### 3.1 Increased thalamo-cortical FCs during REM sleep

**Fig. 2.**
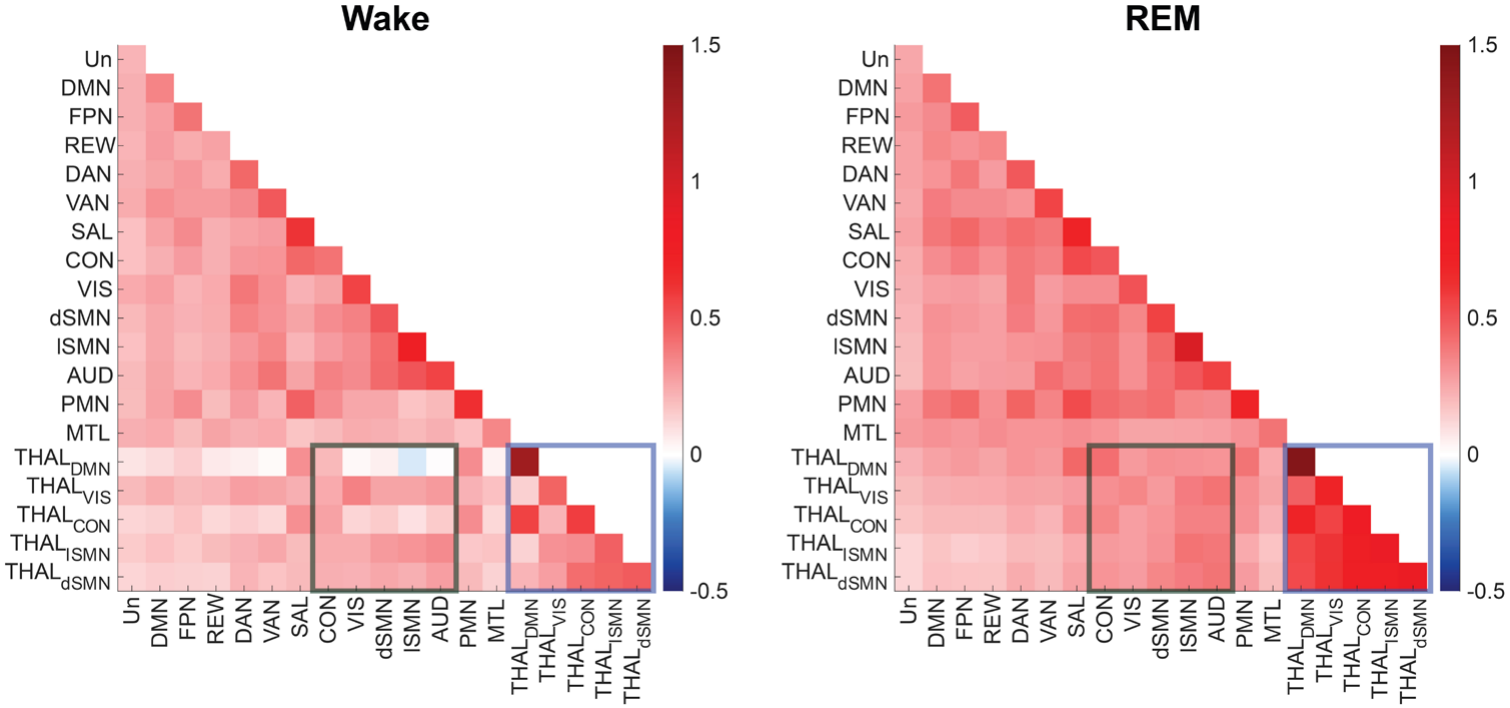
Network FC matrices between 14 cortical networks and five thalamic subnetworks for Wake and REM. The colorbar indicates the Fisher-transformed correlation coefficients, representing between-network FC (off-diagonal) or within-network FC (diagonal). Notes: The blue box highlights FCs among the five thalamic subnetworks; the dark green box highlights FCs between thalamic subnetworks and CON/dSMN/lSMN/AUD. Un, unassigned network; DMN, default mode network; FPN, fronto-parietal network; REW, reward network; DAN/VAN, dorsal/ventral attention network; SAL, salience network; CON, cingulo-opercular network; VIS, visual network; dSMN, dorsal Somatomotor network; lSMN, lateral Somatomotor Network; AUD, auditory network; PMN, parietomedial network; MTL, medial temporal lobe network; FC, functional connection.

Figure 2 displays the FC matrices for Wake and REM, revealing distinct functional organization between these two stages. FC matrices for N1-3 can be found at Supplementary Figure S2. Although REM sleep is often considered to resemble Wake in terms of EEG activity, the FC patterns during REM displayed notable differences from those during Wake, particularly in thalamus-related FCs. As hypothesized, FCs among the five thalamic subnetworks increased during REM, *p* < 0.0001, or FWE corrected (190 comparisons) *p* < 0.05. Additionally, FCs between thalamic subnetworks and sensory-related networks (AUD, VIS, lSMN, dSMN, and CON) were elevated during REM, *p* < 0.0001, or FWE corrected (190 comparisons) *p* < 0.05. Notably, the CON, also referred to as the action-mode network (AMN) (Dosenbach et al., 2024), is involved in action planning, which may be important for sensory integration during dreaming. Similarly, we found increased thalamo-putamen FCs during REM compared to these during Wake, see Supplementary Figure S3.

### 3.2 Increased intra-thalamic connection during REM sleep

**Fig. 3.**
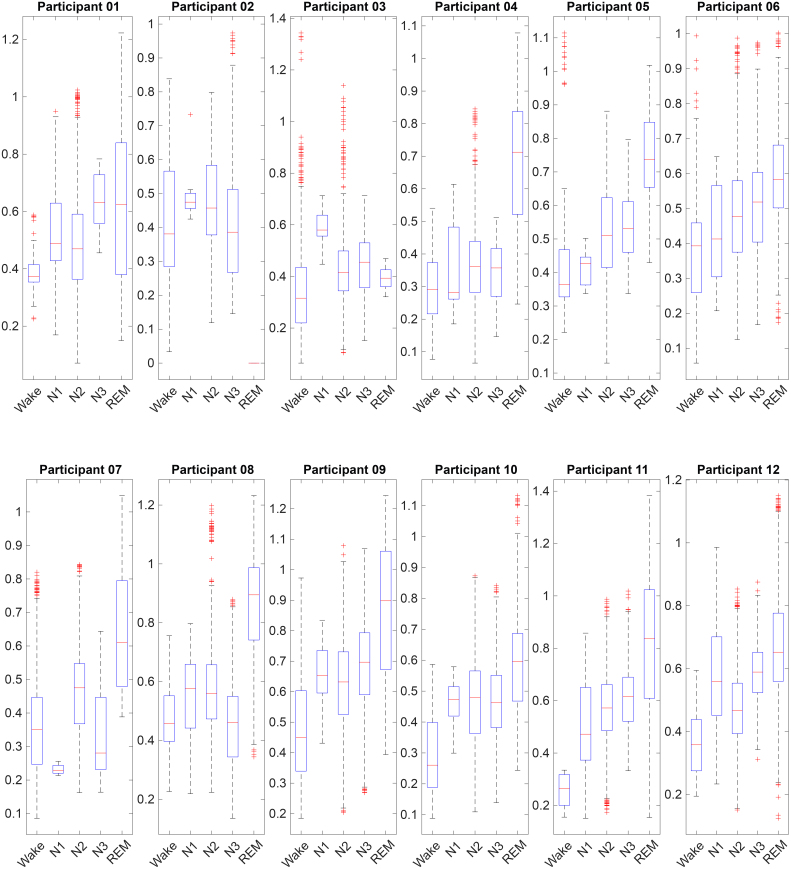
Boxplots of intra-thalamic connection across different sleep stages for each participant. Each box represents one participant. The vertical axis represents fisher-transformed correlation coefficients. Red crosses denote outliers. REM data of Participants 02 were not included due to excessive motion (a red line was shown).

Intra-thalamic connection, calculated as the average FC between each pair of thalamic subnetworks, was higher during REM sleep compared to Wake across all 11 participants with high-quality fMRI data during REM. Additionally, it was the highest among all sleep stages in 9 out of 11 participants (see Fig. 3). Using the Linear Mixed Effects model, we found that intra-thalamic connection during REM sleep was significantly higher than other sleep stages (*p* < 0.0001).

To test the robustness of the intra-thalamic connection result, we conducted two different validations. First, similar results were found in the first night, despite it serving as the adaptation night. See Supplementary Figure S4, increased intra-thalamic connection was found in all seven participants that achieved REM sleep. Second, we found increased intra-thalamic connection during REM sleep compared to Wake even after correction for the global signal, see Supplementary Figure S5.

### 3.3 Higher intra-thalamic connection during phasic REM

**Fig. 4.**
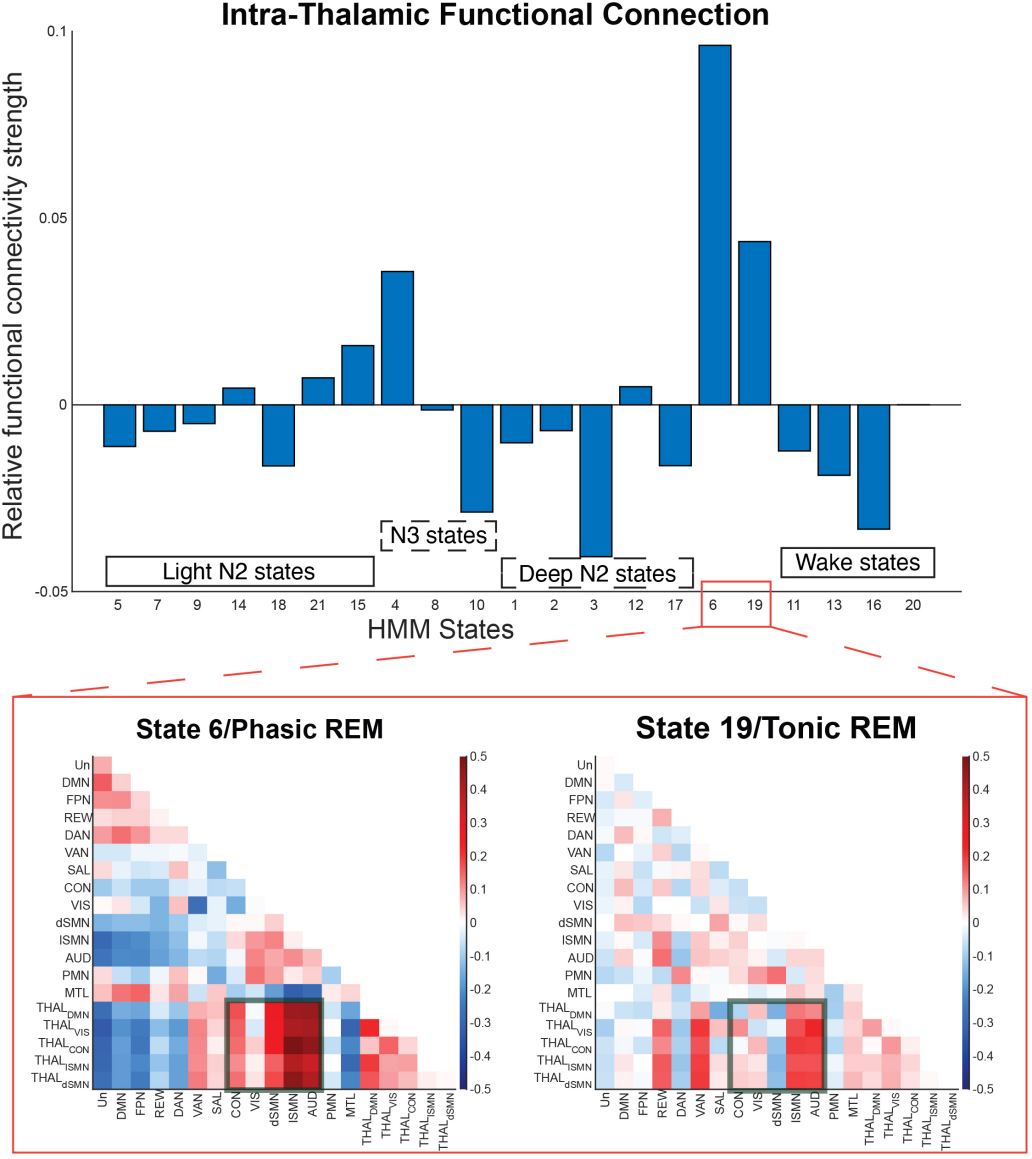
Intra-thalamic and thalamo-cortical FCs between phasic and tonic REM. Top: 21 HMM states were grouped into 5 modules, namely light N2 states, N3 states, Deep N2 states, REM states, and Wake states. Among all the states, Phasic REM (state 6) exhibited the highest intra-thalamic connection among all the states (*p* < 0.001). Bottom: Green boxes highlight that Phasic REM showed higher thalamo-cortical FCs compared to tonic REM (state 19) (*p* < 0.0001). Note: relative FC strength (vertical axis) is as compared to the average FC strength across all 21 HMM states. As different methods were used, the phasic and Tonic REM states were not 100% overlapped with the REM sleep episodes.

Among the 21 HMM-identified sleep states, state 6 (HMM-defined Phasic REM state) exhibited higher intra-thalamic connection compared to all other states (*p* < 0.001), see Figure 4, top panel. Furthermore, FCs between sensory-related networks and thalamic subnetworks were also stronger during Phasic REM than during Tonic REM (HMM state 19, *p* < 0.0001), see Figure 4, bottom panel.

## 4 Discussion

Using a whole-night EEG-fMRI concurrent recording dataset, we discovered that while REM sleep shares certain similarities with Wake, it exhibits significantly elevated intra-thalamic and thalamo-cortical FCs. During Wake, thalamic subnetworks have different connections to cortical networks. However, in REM sleep, all thalamic subnetworks concurrently functionally connect with multiple sensory-related cortical networks. These enhanced thalamus-related FCs may underlie the experience of dreaming, as intra-thalamic and thalamo-cortical FCs were significantly greater during Phasic REM compared to Tonic REM.

This study is one of the first to leverage whole-night EEG-fMRI recordings from non-sleep-deprived participants to investigate the functional organization of the thalamus across all sleep stages, with a specific emphasis on REM sleep. Previously, Chow et al. (2013) conducted an EEG-fMRI study with 11 sleep-deprived participants during late-night hours (02:00–06:00) and, consistent with our findings, observed that the posterior thalamus exhibited stronger FCs to unimodal regions, such as the visual, auditory, and motor cortices, during REM sleep compared to Wake (Chow et al., 2013). Similarly, Hale et al. (2016) reported increased intra-thalamic and thalamo-cortical FCs (between thalamic subdivisions and motor, visual, and somatosensory regions) during the transition from Wake to light sleep stages (N1 and N2) (Hale et al., 2016). Our findings significantly extend these prior results by encompassing all sleep stages and analyzing fine-grained thalamic subnetworks. This was facilitated by a superior spatial resolution compared to previous studies (Chow et al., 2013; Hong et al., 2009; Miyauchi et al., 2009). We reveal that intra-thalamic and thalamo-cortical FCs, including FCs with sensory-related networks beyond primary sensory cortices/networks—such as the cingulo-opercular network (CON) and somatomotor networks (SMN)— increase from Wake to REM sleep.

Results regarding REM-time-locked activations in non-visual sensory cortices have been inconsistent across studies. For example, Miyauchi et al. (2019) studied sleep-deprived participants scanned between 03:00 and 06:00 and observed REM-time-locked activations in the visual cortex, thalamus, putamen, and ACC, but not in non-visual sensory cortices. This may be due to the variations in participants’ sleep deprivation status (Hong et al., 2009; Miyauchi et al., 2009). Conversely, Hong et al. (2009) examined non-sleep-deprived participants and found REM-time-locked activations across a broader range of sensory-related regions, including the visual, motor, and auditory cortices, as well as the thalamus and ACC. Interestingly, a recent study on naturally sleeping pigeons also reported heightened fMRI activation in the thalamus, visual cortex, and non-visual sensory regions during REM sleep compared to NREM sleep (Ungurean et al., 2023).

A recent review suggests that tonic REM serves as an intermediate state between phasic REM and Wake regarding external information processing (Simor et al., 2020). In contrast, phasic REM, characterized by bursts of eye movements linked to ponto-geniculo-occipital (PGO) waves, is associated with vivid dream experiences, absence of external information processing, and increased sensorimotor activity. Consistent with these findings, we show that phasic REM demonstrates greater thalamo-cortical FCs, particularly with somatomotor networks and the action-planning network, compared to tonic REM. Related to our results, a recent intracranial EEG (iEEG) study in epilepsy patients also reported enhanced thalamo-cortical synchronization during phasic REM. Specifically, this study observed increased FCs between the anterior thalamus and scalp EEG signals, particularly in the slow and fast frequency bands, compared to tonic REM (Simor et al., 2021). Given that pontine cholinergic neurons discharge in bursts just before each PGO wave—a process associated with eye movements—it is plausible that these neurons release acetylcholine to the thalamus (Datta & Siwek, 2002; Steriade & McCarley, 2013), which, in turn, activates the limbic system and sensory cortices.

Several limitations of this study are worth noting. First, high-quality REM sleep data were obtained in only 11 participants. Nevertheless, each participant contributed a substantial amount of fMRI data, approximately 16 hours in total. Additionally, our findings were robust, with all 11 participants showing higher intra-thalamic connection during REM sleep compared to Wake. Second, while we used HMM-based classifications of phasic and tonic REM, it is unclear whether these align with the traditional definitions of phasic and tonic REM, which are typically based on the frequency of eye movements. Nonetheless, a recent iEEG study suggests that REM sleep can be subdivided in multiple ways, identifying two distinct sub-stages based on fast versus slow thalamic activity (Bastuji et al., 2024), supporting the potential for alternative classification approaches of the REM sleep. Third, THAL_DMN_ is not maximally functionally connected to the DMN during Wake according to Seitzman et al. (2019), which may be due to not having regressed out the global signal (see Supplement for further discussion).

In summary, this study provides strong evidence that intra-thalamic and thalamo-cortical FCs increase during REM sleep, particularly phasic REM, compared to Wake and NREM stages. These findings suggest that thalamic subregions simultaneously distribute internally generated sensory information to sensory-related cortical networks during vivid dreaming.

## Supplementary Material

Supplementary Material

## Data Availability

The datasets are available at https://openneuro.org/datasets/ds005127/versions/1.0.2. The codes are available at https://github.com/nilsyang/Codes
