# Supplementary material for: Increased intra-thalamic and thalamo-cortical functional connections during human REM sleep: Insights from a two-night EEG-fMRI study
Source: Imaging Neurosci (Camb). 2025 Nov 20;3:IMAG.a.1026. doi: 10.1162/IMAG.a.1026 (PMC12635483; doi:10.1162/IMAG.a.1026)
Supplement: Supplementary Material [file IMAG.a.1026_supp.pdf]

Supplement

$THAL_{DMN}$ is not connected with DMN during Wake.

This discrepancy was likely caused by the differences in preprocessing between Seitzman et al. (2019) the current study. First, they applied global signal regression. Second, they used partial corrections to remove shared variance between subcortical regions, including thalamus, and cortical networks. Third, they have 120 participants while we only have 12, as a larger number of participants might be required to detect a small effect.

Table S1. Number of stable sleep episodes for each participant across different sleep stages.

	P01	P02	P03	P04	P05	P06	P07	P08	P09	P10	P11	P12
**Wake**	2	8	3	6	3	7	12	11	16	5	1	3
**N1**	7	3	3	5	4	15	1	17	8	6	12	18
**N2**	17	17	15	24	7	29	12	23	26	36	20	18
**N3**	3	4	1	6	3	9	3	5	11	9	9	2
**REM**	10	0	1	5	1	1	4	2	3	3	8	7

Table S2. Number of DFCs for each participant across different sleep stages.

	P01	P02	P03	P04	P05	P06	P07	P08	P09	P10	P11	P12
**Wake**	48	194	1018	409	207	198	1079	327	665	216	42	177
**N1**	382	13	41	24	22	228	8	754	88	96	268	675
**N2**	1842	980	581	1583	1344	2109	382	1766	2140	2716	2208	2263
**N3**	83	166	781	179	146	847	247	1404	1036	1199	977	162
**REM**	710	0	21	746	572	427	106	529	408	474	936	1065

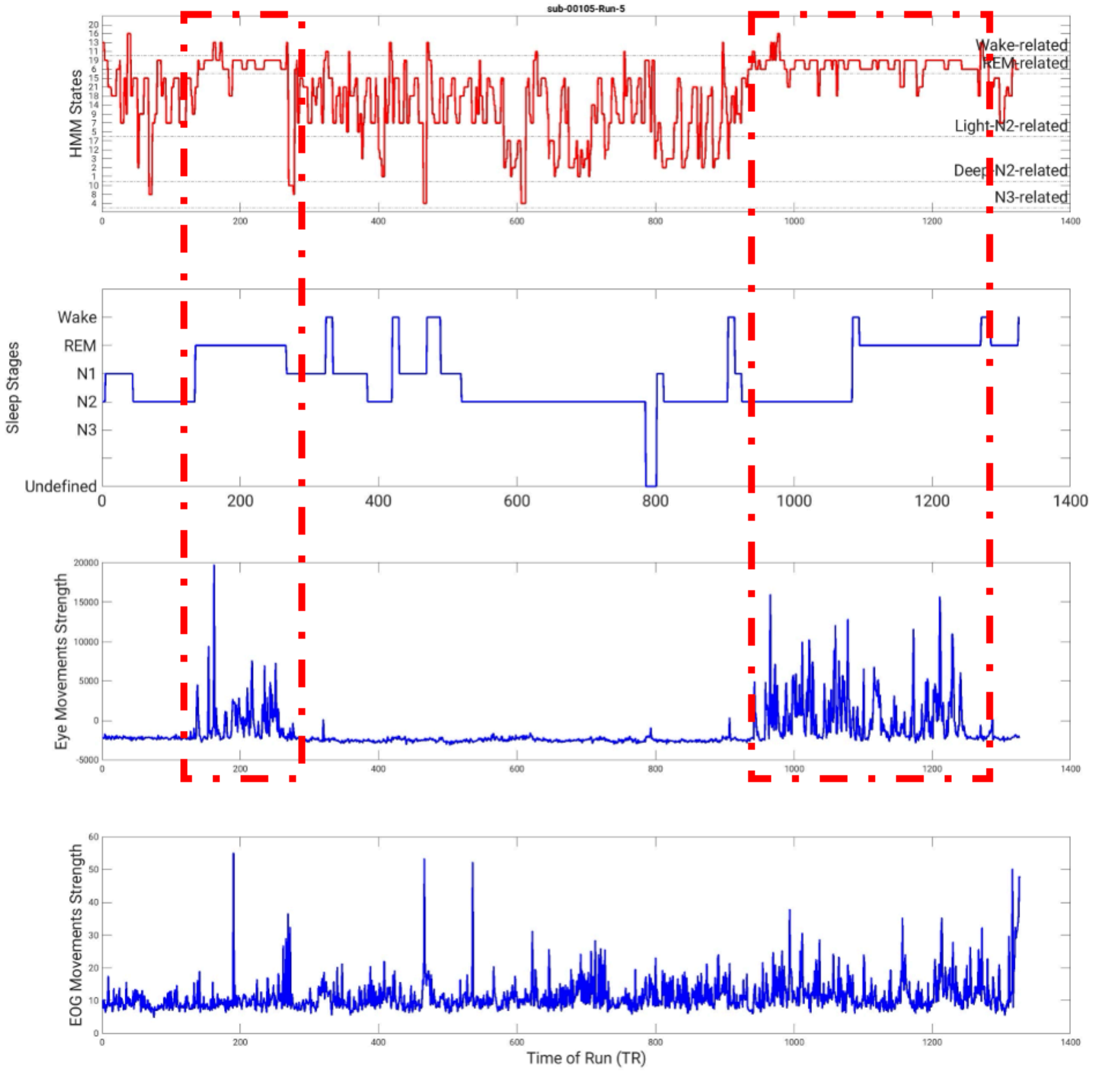

Figure S1. HMM states, sleep scores, Eye movement strengths, and EOG movement strength for an example run. The top panel shows HMM-based states (reordered to align with the second panel, which shows PSG-based sleep stages). The third panel plots eye movements derived from video, and the fourth panel shows the EOG signal. The red boxes highlight time points related to HMM-defined REM states.

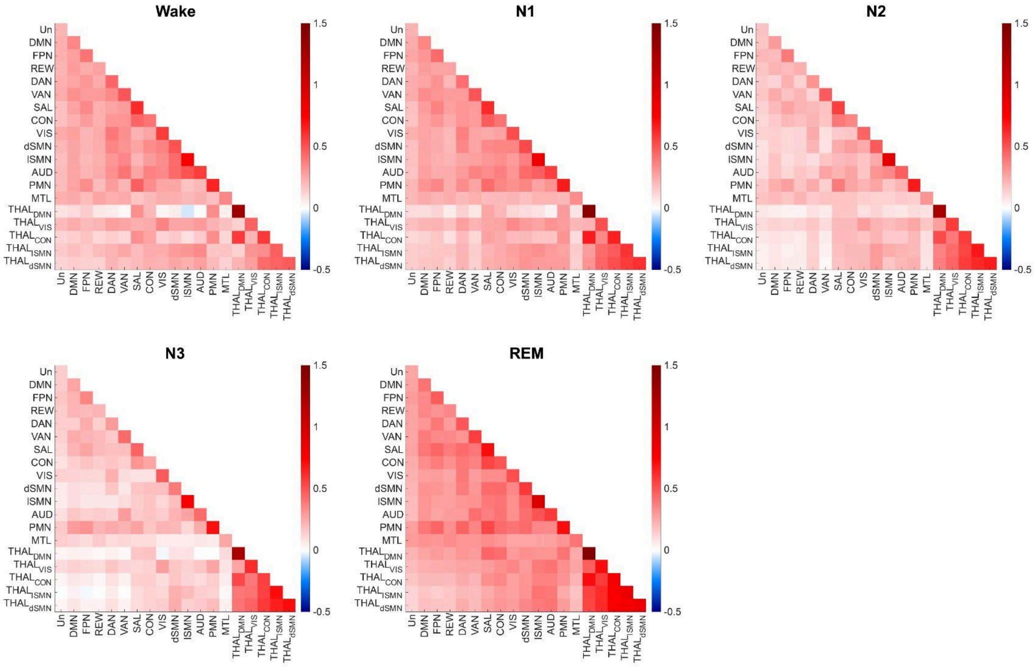

Figure S2. Network FC matrices between 14 cortical networks and five thalamic subnetworks across the five sleep stages. The colorbar indicates the Fisher-transformed correlation coefficients, representing between-network FC (off-diagonal) or within-network FC (diagonal). The top row displays the FC matrix for Wake, N1, and N2 stages. The bottom row displays the FC matrix for N3 and REM. Notes: FC, functional connection.

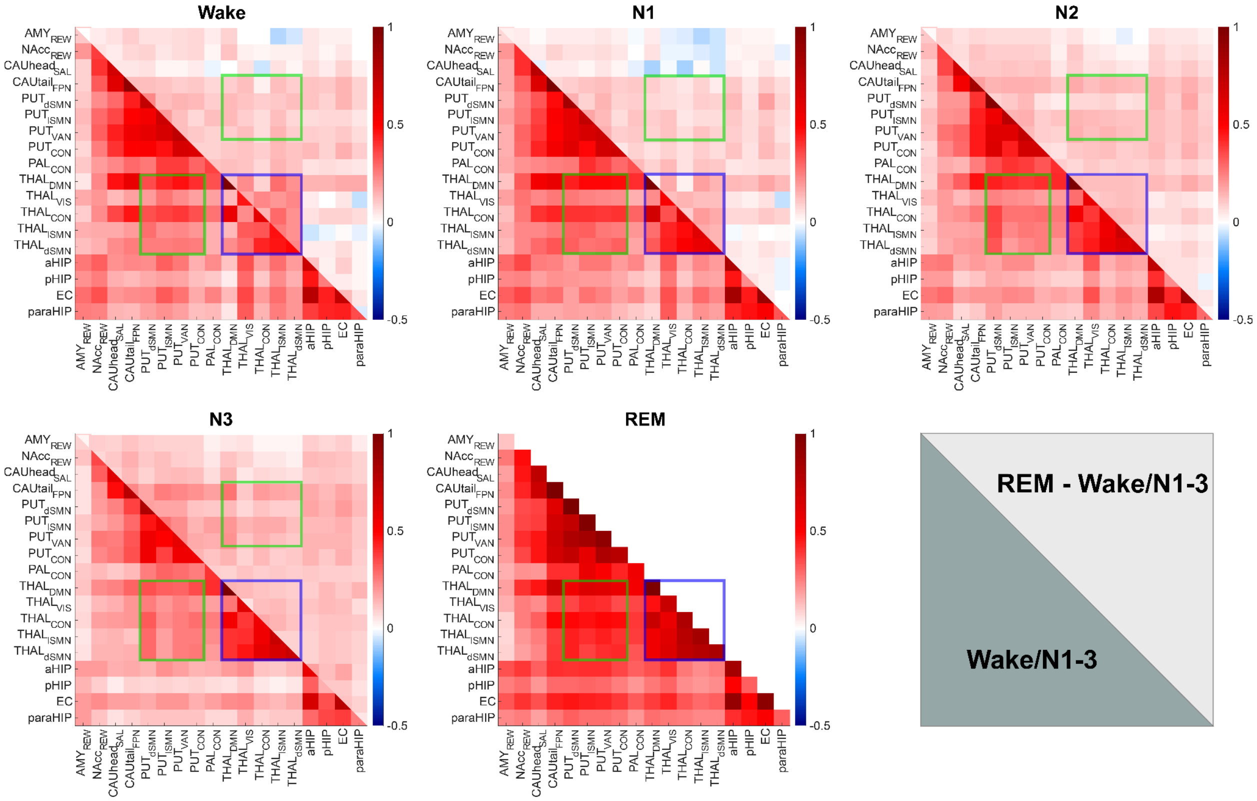

Figure S3. Network FC matrices between subcortical regions and five thalamic subnetworks across the five sleep stages. The colorbar indicates the Fisher-transformed correlation coefficients, representing between-network FC (off-diagonal) or within-network FC (diagonal). Panels **A–D** display the lower triangle as the FC matrix for Wake/N1-3 stages, while the upper triangle highlights the differences in FC between REM and Wake/N1–N3 stages. Panels **E** displays the FC matrix for the REM stage. Notes: The blue box highlights FC among the five thalamic subnetworks; the light green box highlights FCs between thalamic subnetworks and subnetworks of the putamen (PUT). AUD, auditory network; CON, cingulo-opercular network, also known as the action-mode network (AMN); dSMN, dorsal Somatomotor network; lSMN, lateral Somatomotor Network; FC, functional connection.

Figure S4. Boxplots of intra-thalamic connection across different sleep stages for each participant on the **first/adaptation night**. Each box represents one participant. The vertical axis represents fisher-transformed correlation coefficients. Red crosses denote outliers. Five

participants (02, 03, 08, 09, and 11) did not achieve REM sleep on the first night.

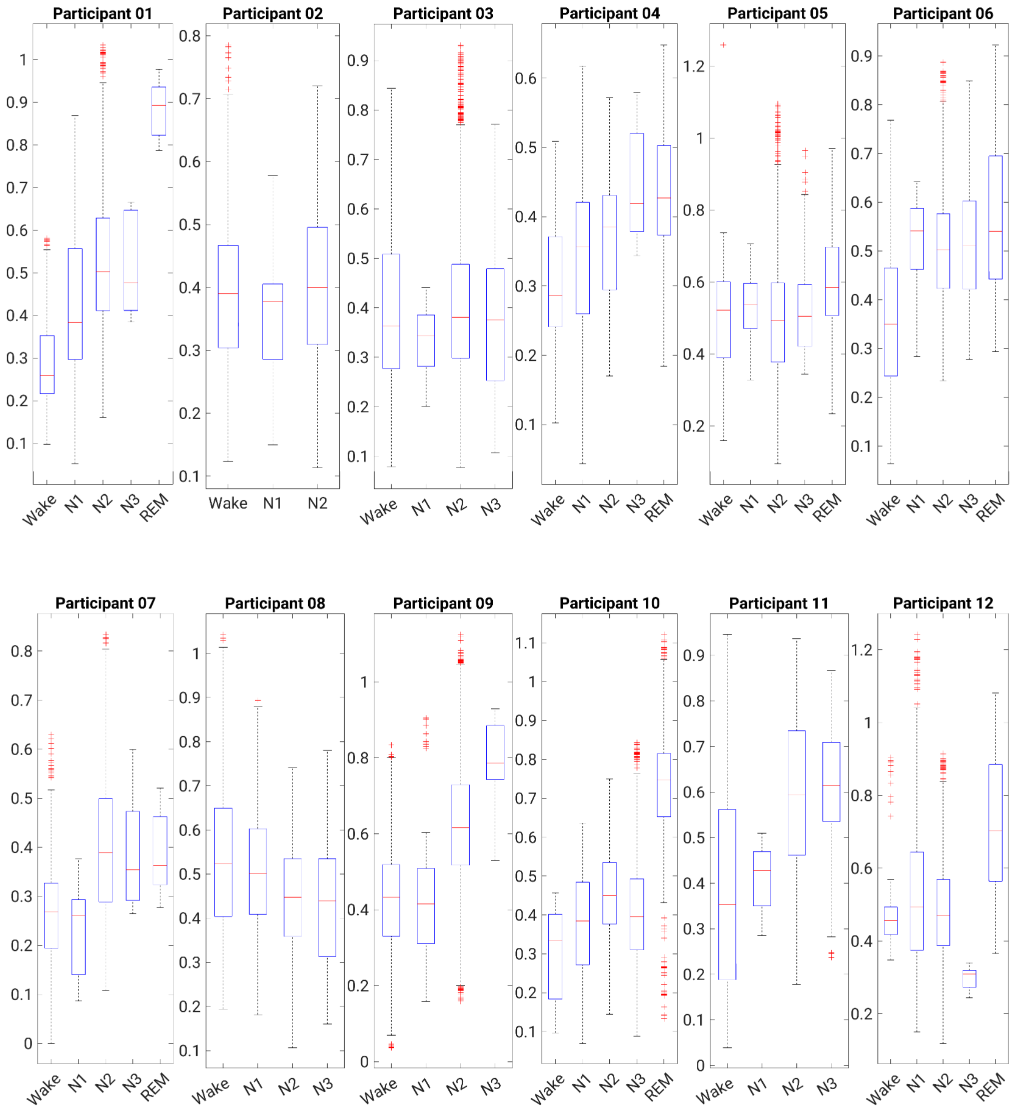

Figure S5. Boxplots of intra-thalamic connection across different sleep stages for each participant. **Global signal regression was applied**. Each box represents one participant. The vertical axis represents fisher-transformed correlation coefficients. Red crosses denote outliers. REM data of Participants 02 was not included due to failed fMRI quality control.

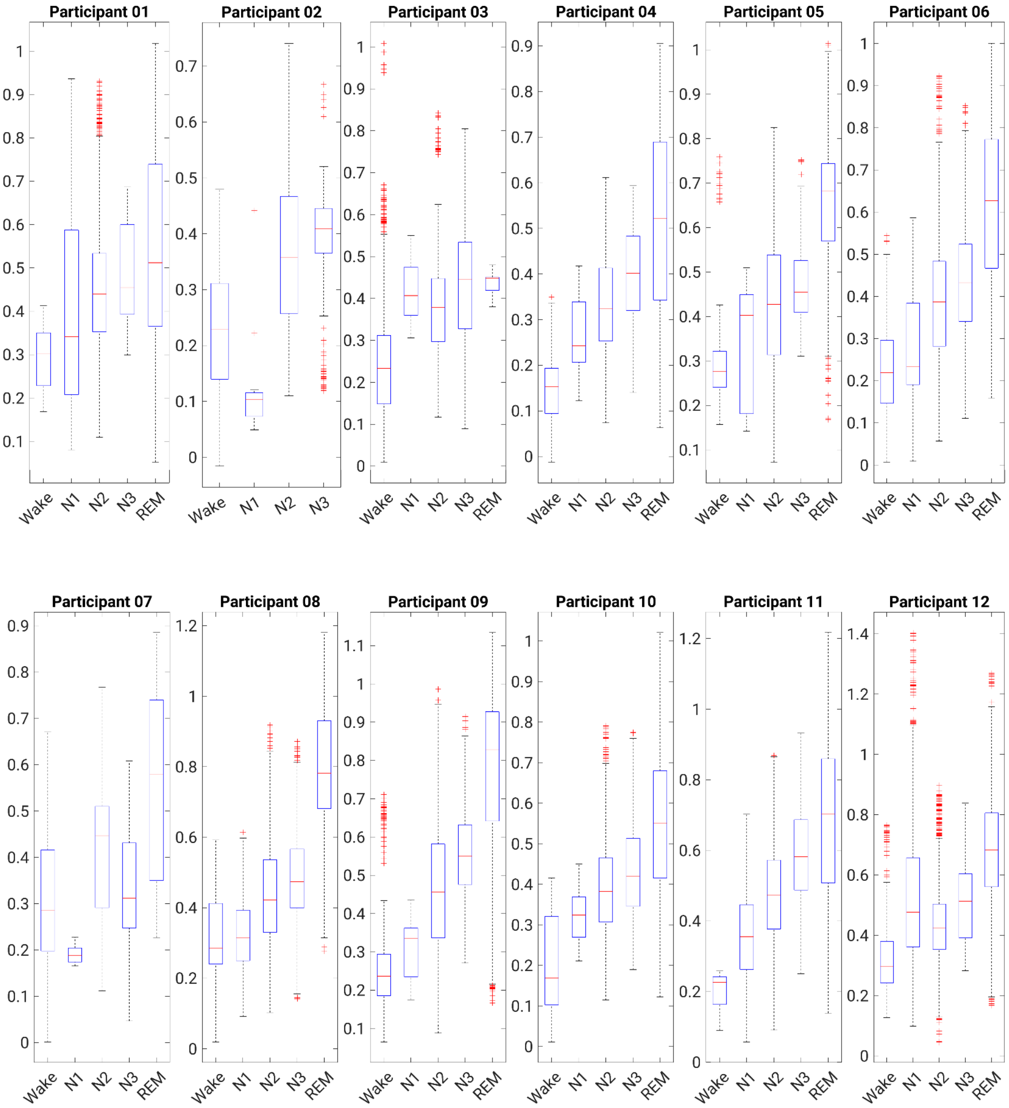

Participant 01
Participant 02
Participant 03
Participant 04
Participant 05
Participant 06
Participant 07
Participant 08
Participant 09
Participant 10
Participant 11
Participant 12
Wake
N1
N2
N3
REM
